# Comparative evaluation of IL-17 and TGF-β expression in tissues of patients with chronic periodontitis and healthy individuals using real-time PCR

**DOI:** 10.15171/japid.2018.002

**Published:** 2018-06-20

**Authors:** Kazem Fatemi, Seyed Abdolrahim Rezaee, Amir Moeintaghavi, Farid Shiezadeh, Golnaz Dadpour, Mohammad Rasoul Asadinezhad, Nahid Nasrabadi

**Affiliations:** ^1^Dental Research Center, Mashhad University of Medical Sciences, Iran; ^2^Immunology Research Center, Inflammation and Inflammatory Diseases Division, Mashhad University of Medical Sciences, Mashhad, Iran; ^3^Maxillofacial Disease Research Center, Mashhad University of Medical Sciences, Mashhad, Iran; ^4^Department of Periodontics, Mashhad University of Medical Sciences, Mashhad, Iran

**Keywords:** Chronic periodontitis, Th17 cells, TGF-beta, regulatory T Cells, reverse transcriptase polymerase chain reaction

## Abstract

**Background:**

The present study aimed to determine the association between periodontal disease and the Th17/Treg balance by examining the genetic expression of IL-17 and TGF-β, which influence incidence and suppression of inflammation.

**Methods:**

In this case-control study, samples were collected in a randomized and task-oriented order. Thirty-seven patients referred to professional periodontology clinics in Mashhad and the Periodontology Department of the Mashhad Dentistry Faculty for periodontal (case) or crown-length (control) surgery was enrolled. IL-17 and TGF-β gene expression indices were measured in tissue samples by real-time polymerase chain reaction.

**Results:**

The IL-17 gene expression index was higher in the case group (2.68±0.91) than in the control group (1.68±0.41), but this difference was not significant. The TGF-β gene expression index was significantly higher in the case group (54.42±7.88) than in the control group (24.12±3.38).

**Conclusion:**

L-17 and TGF-β expression is increased in chronic periodontitis patients, but TGF-β plays a more important role in periodontal inflammation in patients with chronic periodontitis. Further studies of the roles of Th17 and Treg cells are warranted.

## Introduction

Periodontitis is a chronic inflammatory disorder that affects the tooth-supporting tissues. Its pathogenesis involves periodontal pathogens and the host's inflammatory and immune responses. Among immune system components, cytokines play pivotal roles in mediating inflammatory developments and tissue homeostasis underlying periodontitis. Extensive research has demonstrated the expression of and changes in various kinds of cytokines in normal and pathological periodontal tissues.^1^ In response to oral bacteria, the outermost layers of the gingival epithelium produce cytokines, which come into close contact with the commensal oral bacteria.^2^

Clinical parameters such as probing pocket depth, bleeding on probing and tooth mobility are usually used as diagnostic tools for periodontal disease. Host response evaluation is an advanced diagnostic method based on the biochemical or immunological analysis of specific and non-specific mediators considered to contribute to the individual response to periodontal infection.

For many years, the Th1/Th2 paradigm has provided a useful conceptual framework for the investigation of the pathogenesis of periodontitis. However, in periodontitis as in many other inflammatory diseases, the observed role of T cell-mediated immunity does not readily fit this framework. Th17, a recently discovered subset of CD4^+^ T cells, explains many of the discrepancies in the classic Th1/Th2 model. Th17 secretes IL-17, a novel proinflammatory cytokine. The identification of Th17 cells as a novel effector T-cell population compels the re-examination of periodontitis in the context of this new subset and its signature cytokines. Complex interactions between the immune system and periodontal pathogens result in periodontal disease activity. This immunological knowledge is essential for the development of immunomodulatory intervention strategies. Based on this knowledge, we can maximize the protective aspects and minimize the destructive aspects of the periodontal host response.^3^

Th17 cells produce IL-17, IL-22, IL-26 and IL-21. IL-17 is capable of stimulating a variety of cell types to secrete inflammatory mediators, such as IL-1, IL-6, tumor necrosis factor-α, metalloproteinases and chemokines. Th17 cells play a critical role in immune responses against extracellular bacteria. They also participate in the pathogenesis of several autoimmune and inflammatory disorders. IL-17A has been shown to promote the development of osteoclasts in the presence of osteoblasts. Recently, IL-17 has received extensive attention due to its role in periodontal tissue destruction. Moreover, IL-17 has been found to be involved in a number of systemic conditions, including rheumatoid arthritis and several autoimmune disorders.^1^

In appropriate immune responses, Th17 and T regulatory (Treg) cells are balanced precisely to modulate immune reactions. Treg cells produce transforming growth factor (TGF)-β to modulate nearly all the effector arms of immune responses, particularly Th17, thereby preventing harmful inflammatory reactions. A complex balance of interactions between cells and the extracellular matrix (ECM) in human tissues is required to achieve normal homeostasis. Numerous cytokines acting through specific cell-surface receptors are involved in these cooperative interactions. Disturbance of the balance between the cells and ECM can result in disease. One example is the mediating role of TGF-β,^4^ the prototypic member of a superfamily of structurally and functionally related peptides that affect diverse cellular processes.^5^ High or low levels of TGF-β have been associated with numerous diseases, such as atherosclerosis and fibrotic diseases of the kidney, liver and lung. Mutation of the genes for TGF-β, its receptors, or associated intracellular signaling molecules can cause diseases such as cancer and hereditary hemorrhagic telangiectasia. Serum levels of TGF-β and TGF-β mRNA in tissue can be measured and used as diagnostic or prognostic markers for human disease.^4^ The present study aimed to determine the association of periodontal disease with the Th17/Treg balance by examining the genetic expression of IL-17 and TGF-β, which influence the incidence and suppression of inflammation.

## Methods

### Subjects and sample collection

In this case‒control study, samples were collected in a randomized and task-oriented order. Patients with chronic periodontitis, who were referred to the Periodontology Clinic of Mashhad Dental School, were enrolled in the present study. The criteria for periodontitis were attachment loss and minimum probing depth of 6mm or higher in at least eight sites, along with BOP and radiographic bone loss. Data were collected from 37 subjects; 17 subjects were healthy and the remaining 20 had chronic periodontitis. Exclusion criteria were as follows: any systemic diseases or any condition that might interact with periodontal disease, such as diabetes, HIV infection, Papillon-Lefèvre syndrome, any bacterial or viral infection and autoimmune diseases. In patients with periodontitis, biopsies were obtained from the buccal area through a horizontal cut with 5mm distance from the palatal gingiva. In patients with normal gingiva and without periodontitis, similar biopsy of gingival tissue was obtained during crown lengthening surgery. The weight of each biopsy was measured by a sensitive weighing machine. The Board of Research and Ethics Committee of Mashhad University of Medical Sciences, Mashhad, Iran, approved this study and written informed consent was obtained from all the participants.

### RNA extraction and cDNA synthesis

The tissue from each specimen was prepared immediately for total cellular RNA isolation using an RNeasy Mini Kit (Qiagen, Hilden, Germany). After puriﬁcation, RNA was reverse-transcribed to cDNA using random hexamer primers (Fermentas, Germany) according to the manufacturer’s instructions (Table 1).

**Table 1:** Primer and probe sequences used in real-time quantitative reverse transcriptase polymerase chain reactions

**Targeted gene**	**Sequence (5′to3′)**	**Purpose**	**Product Size (bp)**
**IL-17**	GTCAACCTGAACATCCATAACCG	Forward	142
	ACTTTGCCTCCCAGATCACAG	Reverse	
**TGF-β**	GCAAGTGGACATCAACGGGTT	Forward	192
	CGCACGCAGCAGTTCTTCTC	Reverse	
**Beta-2-microglobulin**	TTGTCTTTCAGCAAGGACTGG	Forward	127
	CCACTTAACTATCTTGGGCTGTG	Reverse	
	TCACATGGTTCACACGGCAGGCAT	Probe	

### Real-time polymerase chain reaction

Relative real-time polymerase chain reaction (PCR) assays (SYBR Green method) were performed on the cDNA samples using two standard curves, as described previously.^6^ Briefly, after making six standards using 10-fold serial dilutions of a concentrated sample for the gene of interest and the reference gene (beta-2 microglobulin [β2M]), the reactions for standards and samples were adjusted in Universal Master Mix (Takara, Otsu Shiga, Japan) and performed with a Q6000 Rotor Gene device (Qiagen). Standard curves were then produced for the gene of interest and reference gene, and the unknown results were then analyzed using Rotor Gene 6000 software program (Qiagen) for the quantification of gene expression. The relative quantities of mRNA copies for IL-17 and TGF-β were then normalized to the relative quantity of β2M mRNA and reported as Gene Expression Indices (GEIs). By comparing the mean GEIs of the control and target groups, the fold change in expression of these genes was calculated and statistically analyzed.

### Statistical analysis

Data were analyzed using SPSS 13 (SPSS Inc., Chicago, IL, USA). As the distributions of IL-17 and TGF-βdata were not normal according to the Kolmogorov–Smirnov test, differences in these data between the two groups were analyzed with the non-parametric Mann–Whitney test. Statistical significance was set at P<0.05.

## Results

Gingival tissue samples were collected from a total of 37 subjects: 20 patients with chronic periodontal disease and 17 healthy individuals. The mean GEI of IL-17 was higher in the case group (2.68±0.91) compared to the control group (1.68±0.41), but the difference was not significant (Table 2). The mean GEI of TGF-βwas significantly higher in the case group (54.42±7.88) compared to the control group (24.12±3.38, P=0.005).

**Table 2:** Gene expression indices of IL-17 and TGF-β in gingival tissue samples

**Group**	**N**	**GEI of IL-17**	**P-value**	**GEI of TGF-β**	**P-value**
**Case**	20	2.68±0.91	0.926	54.42±7.78	0.005
**Control**	17	1.68±0.41		24.12±3.38	

Data are presented as mean ± standard deviation of the mean. GEI = gene expression index

## Discussion

Recent advances in cellular and molecular biology have improved our understanding of the mechanisms of inflammatory and immune responses in many infectious diseases. Cytokines, as soluble mediators produced by various inflammatory and structural cells, play central roles in the pathogenesis of most of these diseases, including periodontal disease.^7^ However, studies conducted to elucidate the cytokine networks involved in chronic periodontitis have produced contradictory results. Periodontal disease is caused by bacteria in the dental plaque, which are responsible for the progressive form of the disease. Interestingly, some individuals have these specific microorganisms, but do not show evidence of disease progression. Thus, although periodontal bacteria are the major etiological agents in periodontitis, the host immune response to these bacteria seems to play a pivotal role.^8^

In the present study, the IL-17 GEI did not differ significantly between the case and control groups, although it was higher in the former. Several studies have documented the role of IL-17 in periodontal diseases.^9,10^ Fu et al^1^ showed that the removal of dental plaque resulted in improvements in clinical parameters, with GCF IL-17 decreasing to control levels while GCF interferon-γ and IL-10 levels remained unchanged. They concluded that the decrease in IL-17 in GCF from patients showing resolution of periodontitis suggests that IL-17 is involved in the periodontal inflammatory process. Kadkhodazadeh et al^11^ studied the prominent impacts of genetic factors on periodontitis and IL-17 levels in blood samples. They found that IL-17 polymorphism played significant roles in chronic periodontitis and peri-implantitis. Shaker et al^12^ evaluated IL-17 in GCF from patients with generalized chronic periodontitis (GCP) and generalized aggressive periodontitis (GAgP) in relation to periodontopathic bacteria. They found that the total amount of IL-17 was significantly greater in the GAgP group than in the GCP and control groups, which can explain our results to some extent. In our study, the case group received initial treatment for periodontal disease before undergoing surgery and tissue sample collection; in a study by Shaker et al,^12^ patients did not received treatment at the time of sample collection. Our approach might have led to the lack of a significant difference in the IL-17 level between the case and control groups. However, Rohaninasab et al^13^ showed that the effect of the first phase of periodontal therapy on IL-17 and IL-23 levels in GCF in patients with severe periodontitis and control subjects was significant. They reported significant differences in IL-17 viscosity between groups before and after therapy, which is not consistent with our results. This discrepancy might be related to the IL-17 assays and samples used; we used RT-PCR to assay IL-17 in the gingival tissue, whereas Rohaninasab et al^13^ used ELISA to determine IL-17 concentrations in GCF collected with PerioPaper. Moreover, they collected samples from treated sites 4 weeks after the first phase of periodontal therapy.^13^

In the present study, the TGF-β GEI was significantly higher in the case group compared to the control group. Khalaf et al^14^ recently determined cytokine profiles in serum, saliva and GCF from patients with periodontitis and healthy controls. They reported that patients with periodontitis exhibited greater numbers of periodontal pathogens, significantly altered immune responses and significantly elevated TGF-β1 levels. Their results indicate that *Porphyromonasgingivalis* is a major contributor to the altered immune responses and pathology of periodontitis.^14^ As TGF-β has been implicated in differentially regulated gene expression in gingival fibroblasts, Ohshima et al^15^ hypothesized that TGF-β signaling would be activated in periodontitis-affected gingiva, along with enhanced collagen degradation. They concluded that TGF-β signaling is involved in fibroblast–epithelial cell interaction in periodontitis. Another study showed that TGF-β1, which is largely involved in tissue regeneration and remodeling, is regulated in chronic periodontitis.^16^ n the same study, the gingival expression of connective tissue growth factor (CTGF or CCN2), a TGF-β1–upregulated gene, was determined in patients with periodontitis. The authors quantified the expression of CTGF and TGFβ1 mRNAs using real-time PCR. Both expression levels were significantly higher in individuals with periodontitis compared with those without periodontitis.^16^ Another study was conducted to evaluate the effect of surgical flaps on the concentrations of IL-1β and TGF-β in the GCF of patients with moderate to severe chronic periodontitis.^17^ Flap surgery led to a significant reduction of the IL-1β concentration. The authors suggested that reduction in the TGF-β1 concentration after treatment was due to the removal of inflammatory stimulants.^17^ In the current study, as tissue samples were collected during surgery from patients who had received the first phase of periodontal therapy, lower levels of tissue inflammation and secreted IL-17 and other pro-inflammatory cytokines were observed.

In conclusion, our results showed that IL-17 and TGF-β levels were higher in patients with periodontal disease, but TGF-βplays a more important role in periodontal inflammation in patients with chronic periodontitis. Further studies on the roles of Th17 and Treg cells in periodontal disease are warranted.

## Competing interests

The authors declare no conflict of interests.

## Ethics approval

All procedures performed in this study involving human participants were in accordance with the ethical standards of the institutional research committee and with the 1964 Helsinki declaration and its later amendments or comparable ethical standards(Ethical registration number: IR.mums.D.REC.1394.54)
